# Genome-Wide Identification Analysis of the MAPKKK Gene Family in Cotton and Its Role in Development and Stress Response

**DOI:** 10.3390/ijms27021124

**Published:** 2026-01-22

**Authors:** Yahui Deng, Nan Zhao, Shuo Ning, Yifan Wang, Weiran Wang, Meng Wang, Zixin Zhou, Yaohua Li, Caixia Li, Lingfang Ran, Jiahui Zhu, Zhiqing Liu, Jing Yang, Alifu Aierxi, Jie Kong, Aixing Gu, Jianping Li

**Affiliations:** 1 Xinjiang Key Laboratory of Cotton Genetic Improvement and Intelligent Production/National Cotton Engineering Technology Research Center, Cotton Research Institute of Xinjiang Uyghur Autonomous Region Academy of Agricultural Sciences, Urumqi 830091, China; huiyad0330@163.com (Y.D.); nan_zhao@cau.edu.cn (N.Z.);; 2 College of Agronomy, Xinjiang Agricultural University, 311 Nongda East Road, Urumqi 830052, China

**Keywords:** upland cotton, sea island cotton, mitogen-activated protein kinase kinase kinase, modular functional diversification, stress defense

## Abstract

Mitogen-activated protein kinase kinase kinases (MAPKKKs) are pivotal upstream regulators of MAPK cascades, integrating signals that coordinate plant development and stress responses. However, the specific functions of MAPKKKs, particularly within the MEKK subfamily, in mediating cotton resistance to Verticillium wilt and Fusarium wilt remain poorly characterized. To address this, we conducted a systematic, cross-species analysis of the MAPKKK family in four key cotton species: *Gossypium arboreum*, *Gossypium barbadense*, *Gossypium hirsutum*, and *Gossypium raimondii*. Genome-wide identification and phylogenetic analysis revealed 660 MAPKKK genes, classifying them into the MEKK, Raf, and ZIK subfamilies. Evolutionary analysis indicated that Whole-Genome Duplication (WGD) events were the primary driver of family expansion. Promoter *cis*-element and Gene Ontology (GO) enrichment analyses implicated these genes in hormone signaling and stress adaptation. Expression profiling demonstrated functional modularity, with distinct members responding specifically to cold stress or cooperatively to drought and salt stresses. Upon pathogen infection, members diverged into regulatory modules associated with immune homeostasis, tissue-specific defense, and core signaling potentially governing systemic acquired resistance (SAR). The temporal expression patterns of core candidate genes were validated by qRT-PCR. This study provides, for the first time, a comprehensive evolutionary and functional framework for the MEKK subfamily within the cotton MAPKKK family. It reveals the conserved and divergent roles of this subfamily in stress adaptation and identifies key candidate genes for breeding disease-resistant cotton varieties.

## 1. Introduction

Plant diseases, particularly those caused by fungal pathogens, represent one of the most severe threats to global food security and sustainable agriculture. Vascular wilt diseases, incited by soil-borne fungi such as *Fusarium oxysporum* and *Verticillium dahliae*, inflict devastating yield losses by colonizing the plant vascular system and disrupting the transport of water and nutrients [1]. The challenge in managing these pathogens is compounded by their stealthy infection strategies, including long-term soil survival, asymptomatic spread, and systemic colonization, which render early detection and effective control exceedingly difficult. The economic impact is staggering; in cotton alone, annual yield losses due to *Verticillium wilt* are estimated to exceed 10%, with complete crop failure occurring in severe outbreaks [2]. Furthermore, the overreliance on chemical fungicides has not only accelerated the evolution of pathogen resistance but also raised significant environmental and human health concerns [3]. Consequently, deciphering plant immunity mechanisms to develop sustainable disease management strategies has become an urgent priority in modern agriculture [4].

In the co-evolutionary arms race with pathogens, plants have evolved a sophisticated, multi-layered immune system [5]. Pattern-triggered immunity (PTI) and effector-triggered immunity (ETI) constitute the two primary layers of this defense network, relying on conserved signaling cascades for signal transduction [6]. Among these, the mitogen-activated protein kinase (MAPK) cascades are highly conserved signaling modules in eukaryotes [7]. A typical MAPK cascade consists of three sequentially acting kinases: MAPK kinase kinase (MAPKKK or MEKK), MAPK kinase (MKK or MAP2K), and MAPK (MPK) [8]. This relay amplifies and transmits stress signals through sequential phosphorylation, ultimately activating defense responses such as defense gene expression, reactive oxygen species (ROS) burst, and hormonal signaling networks, playing a central role in plant responses to both biotic and abiotic stresses [9].

The functional conservation and diversity of the MAPKKK family have been extensively documented in model plants and crops [10]. Arabidopsis MAPK3 or MPK3 and MPK6 play important signaling roles in plant immunity and growth/development. MKK4 and MKK5 function redundantly upstream of MPK3 and MPK6 in these processes. YODA (YDA), also known as MAPKKK4, is upstream of MKK4/MKK5 and forms a complete MAPK cascade (YDA–MKK4/MKK5–MPK3/MPK6) in regulating plant growth and development. In plant immunity, MAPKKK3 and MAPKKK5 function redundantly upstream of the same MKK4/MKK5–MPK3/MPK6 module [11]. In tomato, MEKK subfamily members, including *SlMKK2/4* enhance resistance to *Botrytis cinerea* via jasmonic acid/ethylene signaling pathways. These findings underscore the conserved, pivotal role of MAPKKKs, particularly the MEKK subfamily, in plant immune signaling, providing a crucial reference for investigating MAPKKK mechanisms in cotton [12,13]. What is more, in rice, Raf-like MAPKKK genes such as *DSM1* contribute to drought stress tolerance, while ILA1 regulates leaf angle and sclerenchyma development, illustrating functional diversification within the family [14].

Cotton (*Gossypium* spp.), a vital global source of natural fiber and oilseed, is susceptible to various fungal pathogens throughout its lifecycle. *Verticillium wilt*, caused by *V. dahliae*, and *Fusarium wilt*, caused by *F. oxysporum* f. sp. *vasinfectum*, are collectively regarded as the cancer of cotton [15]. *V. dahliae* invades through the roots, colonizes the vasculature, and secretes cell-wall-degrading enzymes, leading to wilting and plant death [16]. Its microsclerotia can persist in soil for decades, making conventional control methods largely ineffective. *F. oxysporum* is most virulent at 25–30 °C, causing seedling mortality up to 37% and vascular blackening in mature plants [17]. Therefore, given the persistent threat of these soil-borne vascular wilt diseases and their devastating agronomic impact, deciphering the molecular mechanisms of cotton-pathogen interactions—particularly immune signaling networks—is critical to developing durable disease resistance strategies [18]. In cotton, MAPK cascades have been implicated in resistance to both *Verticillium* and *Fusarium wilts*. Among the 114 MAPKKK family members identified in *Gossypium raimondii* [19]. We also found that *GhMAPKKK2* was shown to regulate *Verticillium wilt* resistance via the MAPK pathway [20]. The *GhMKK6*-mediated cascade, finely tuned by miRNAs and MAPK scaffold proteins, plays a critical role in *Fusarium wilt* resistance [21]. Furthermore, the introgression of an NLR-type immune receptor from *G. arboreum* into upland cotton (*G. hirsutum*) has been significantly associated with enhanced *Verticillium wilt* resistance, offering clues for exploring the synergistic network between MAPKKKs and NLR receptors [22].

Despite the well-established role of MAPK pathways in model plant immunity, significant knowledge gaps remain regarding the upstream regulatory mechanisms of the MAPK signaling network in allopolyploid cotton [23,24,25]. As an allotetraploid, upland cotton possesses an expanded MAPKKK family, with 218 members identified, far exceeding the number found in its diploid progenitors. This polyploidization-driven expansion, coupled with potential expression and functional asymmetry between homoeologous genes from the A and D subgenomes, may confer enhanced stress adaptability. However, a comprehensive, multi-omics investigation into the evolutionary history, regulatory hierarchy, and functional differentiation of the MAPKKK family, particularly the MEKK subfamily, in the context of modular defense against vascular wilt pathogens in cotton is still lacking [26,27].

To address these gaps, this study employs a multi-omics approach utilizing updated genome data from *G. hirsutum*, *G. barbadense*, *G. arboreum*, and *G. raimondii*, along with transcriptome profiles under diverse biotic and abiotic stresses. Our objectives are to: (1) systematically identify MAPKKK family members and analyze their evolutionary history, gene structure, and conserved motifs; (2) characterize the co-expression patterns of GhMAPKKKs under multiple stresses; (3) elucidate the expression dynamics of MEKK subfamily members during different stages of pathogen infection; (4) screen for key candidate genes associated with vascular wilt resistance; and (5) explore the regulatory roles of GhMAPKKKs within the immune signaling network. This research innovatively provides the first comprehensive overview of the evolution and functional landscape of the MAPKKK family in vascular wilt resistance in a polyploid crop. The findings will deepen our understanding of plant immune signaling networks and provide novel targets and genetic resources for molecular breeding of wilt disease-resistant cotton varieties, thereby advancing strategies for genetic and eco-friendly crop disease control.

## 2. Results

### 2.1. Identification and Characterization of MAPKKK Gene Family in Gossypium Species

A comprehensive genome-wide analysis of protein sequences led to the identification of 218 *G. hirsutum* MAPKKKs (GhMAPKKKs), 98 *G. raimondii* MAPKKKs (GrMAPKKKs), 114 *G. arboreum* MAPKKKs (GaMAPKKKs), and 230 *G. barbadense* MAPKKKs (GbMAPKKKs), respectively (Table 1). The identified MAPKKK members were classified into three primary subfamilies: MEKK, Raf, and ZIK. In the allotetraploid *G. hirsutum* (AD_1_), the 218 GhMAPKKKs included 58 MEKK, 132 Raf, and 28 ZIK members. In the allotetraploid *G. barbadense* (AD_2_), the 230 GbMAPKKKs were distributed as 60 MEKK, 142 Raf, and 28 ZIK members. In the diploid *G. arboreum* (A_2_), the 114 GaMAPKKKs comprised 30 MEKK, 69 Raf, and 15 ZIK members. In the diploid *G. raimondii* (D_5_), the 98 GrMAPKKKs consisted of 29 MEKK, 55 Raf, and 14 ZIK members. The total number of members in the allotetraploid species (*G. barbadense* and *G. hirsutum*) is greater than the simple sum of their diploid progenitors (*G. arboreum* and *G. raimondii*), suggesting potential gene loss or gain following polyploidization. Bioinformatic analysis of the physicochemical properties revealed considerable diversity among the encoded proteins. The protein length varied from 270 to 1583 amino acids, corresponding to molecular weights ranging from approximately 30.71 to 169.31 kDa. The theoretical isoelectric point (pI) spanned a wide range from 4.20 to 9.88. Notably, the majority of the MAPKKK proteins in all four species had a theoretical pI greater than 7.00, indicating that the MAPKKK family in cotton is predominantly basic in nature (Table 1; Supplementary Table S1). This comprehensive identification and characterization provide a foundation for subsequent evolutionary and functional analyses of the MAPKKK family in cotton.

### 2.2. Genomic Distribution of MAPKKK Genes in Gossypium Species

**Figure 1:**
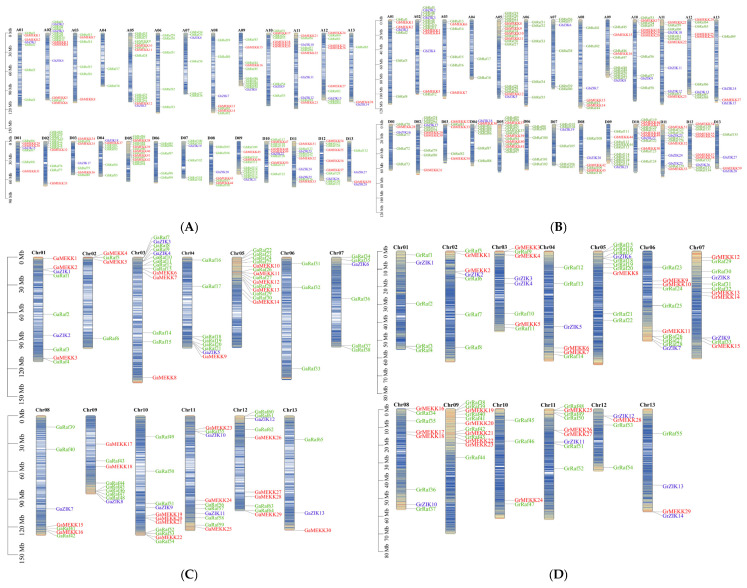
Genomic distribution of MAPKKK genes in four *Gossypium* species. (**A**) Chromosomal locations of MAPKKK genes in *G. hirsutum*. (**B**) Chromosomal locations of MAPKKK genes in *G. barbadense*. (**C**) Chromosomal locations of MAPKKK genes in *G. arboreum*. (**D**) Chromosomal locations of MAPKKK genes in *G. raimondii*. The chromosome numbers are indicated at the top of each chromosome. The scale bar on the left represents chromosome length (Megabases, Mb). This distribution map was generated using TBtools (v1.098).

To gain a comprehensive understanding of the genomic organization and potential duplication events, we mapped the physical locations of all identified MAPKKKs onto the chromosomes of the four cotton species. Our analysis revealed that the MAPKKKs are distributed across all chromosomes but exhibit a non-random, uneven distribution pattern in each species (Figure 1). In the allotetraploid species *G. hirsutum*, the 218 GhMAPKKKs were dispersed across all 26 chromosomes, with gene number per chromosome ranging from 2 to 15 (Figure 1A). Notably, A02 and A05 carried the highest number of genes (15). In contrast, chromosome A04 was found to be particularly sparse, containing only 2 MAPKKKs, both of which belonged to the Raf subfamily. A comparable distribution pattern was observed in the other allotetraploid, *G. barbadense* (AD_2_) (Figure 1B). Its 230 GbMAPKKKs were also unevenly distributed. Chromosome A05 was the most gene-rich, with 18 members, followed by D05 with 14 genes. Mirroring the finding in *G. hirsutum*, A04 in *G. barbadense* also contained the fewest genes (2), exclusively from the Raf subfamily. In the diploid *G. arboreum*, the 114 GaMAPKKKs are located on all 13 chromosomes, but the gene number per chromosome varied significantly, ranging from 3 to 14 (Figure 1C). Chr03 and Chr05 harbored the highest density of MAPKKK genes, with 14 genes each, while Chr06 and Chr13 contained the fewest, with only 3 genes each. A parallel analysis in the other diploid progenitor, *G. raimondii*, showed a similar uneven distribution for its 98 GrMAPKKKs (Figure 1D). Chr09 contained the maximum number of genes (12), whereas Chr10, Chr12, and Chr13 had the lowest density, with only 4 genes each. Collectively, these results demonstrate an asymmetric distribution of MAPKKK genes across the cotton genomes. The uneven gene density across different chromosomes and chromosomal regions likely reflects historical genomic rearrangements, gene loss, and duplication.

### 2.3. Phylogenetic Analysis of the MAPKKK Gene Family in Gossypium

**Figure 2:**
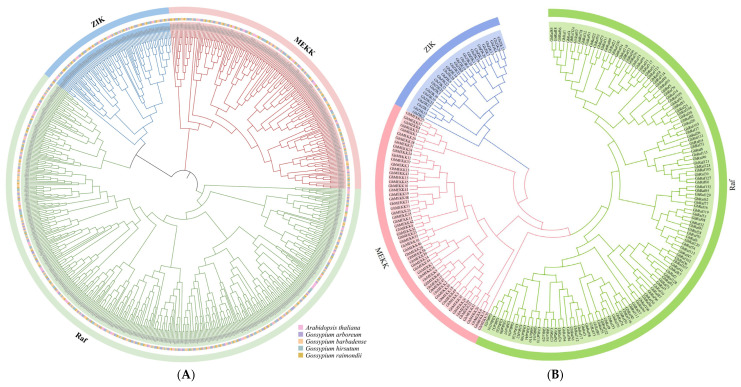
Phylogenetic tree of the MAPKKK gene family in four cotton species and *Arabidopsis*. (**A**) Phylogenetic relationships of the MAPKKK gene family in four cotton species. (**B**) Phylogenetic relationships of the MAPKKK gene family in *G. hirsutum*.

To elucidate the evolutionary relationships of the MAPKKK gene family, a comprehensive phylogenetic tree was constructed using protein sequences from the four cotton species and *A. thaliana.* The phylogenetic analysis robustly classified all MAPKKK proteins into three conserved subfamilies: MEKK, Raf, and ZIK (Figure 2). A striking disparity in subfamily size was observed. The Raf subfamily was the most expansive, comprising 398 members and accounting for approximately 60.30% of the total, whereas the ZIK subfamily was the smallest, with only 85 identified members (approximately 12.88%). This significant numerical difference suggests that the Raf subfamily may have undergone more frequent gene duplication events during evolution, potentially leading to the acquisition of more diverse biological functions. The topology of the phylogenetic tree further revealed that the allotetraploid cottons originated from the hybridization and subsequent polyploidization of A and D genome diploid ancestors. Furthermore, orthologous genes from *G. hirsutum* and *G. barbadense* consistently formed tightly clustered sister branches, which offers molecular-level confirmation that these two species share common ancestors. Although some evolutionary clades contained a limited number of members, their stable conservation throughout the long evolutionary history of cotton implies they may perform indispensable, fundamental biological roles.

### 2.4. Gene Structure and Conserved Motif Analysis of the MAPKKK Family in G. hirsutum

Based on phylogenetic analysis, this study classified the MAPKKK gene family members in upland cotton into three evolutionarily distinct subfamilies: ZIK, MEKK, and Raf. Each subfamily exhibits distinct patterns in conserved motif composition and gene structure, providing a structural basis for their functional divergence.

**Figure 3:**
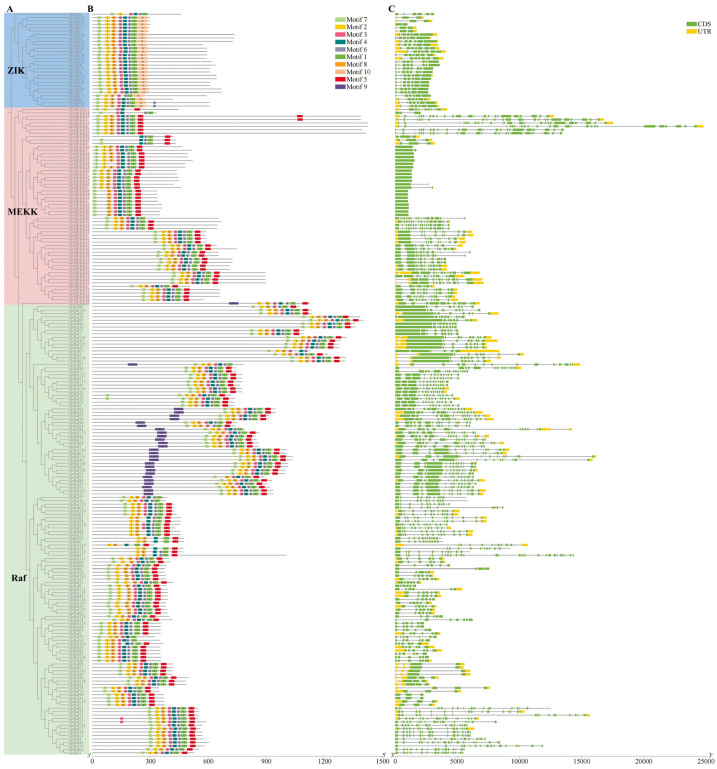
Phylogenetic relationship, conserved motifs, and gene structure of MAPKKK genes in *G. hirsutum*. (**A**) Phylogenetic tree of MAPKKK proteins constructed using the Maximum Likelihood method. The tree classifies all members into three distinct subfamilies (MEKK, Raf, and ZIK), indicated by different colored backgrounds. (**B**) Distribution of conserved motifs in MAPKKK proteins. A total of ten motifs were identified using the MEME suite, with each motif represented by a unique color. The schematic diagram shows the type, order, and relative position of motifs in each protein. (**C**) Exon-intron structure of MAPKKK genes. Green rectangles represent coding sequence (CDS) and yellow rectangles denote untranslated regions (UTRs). The phylogenetic order of genes is consistent across all panels.

In terms of motif composition, the ZIK subfamily displays a highly streamlined profile, with over 60% of its members containing only 6–7 core motifs, yet all retain the essential catalytic motifs (Motifs 1–3). The MEKK subfamily features a conserved set of 9 motifs, including characteristic ones such as Motif 5, which possesses a calmodulin-binding feature, and Motif 9, containing a leucine zipper structure, potentially mediating specific protein interactions. The Raf subfamily shows the greatest structural diversity; approximately 26.5% of its members retain a complete motif set, while a significant portion exhibits the absence of characteristic motifs (Motif 10). The structural complexity of the MEKK subfamily lies between the other two (Figure 3). These differences align with their respective motif conservation patterns, collectively forming a multi-layered and refined potential regulatory foundation for this gene family.

### 2.5. Synteny Analysis of the MAPKKK Gene Family in G. hirsutum

**Figure 4:**
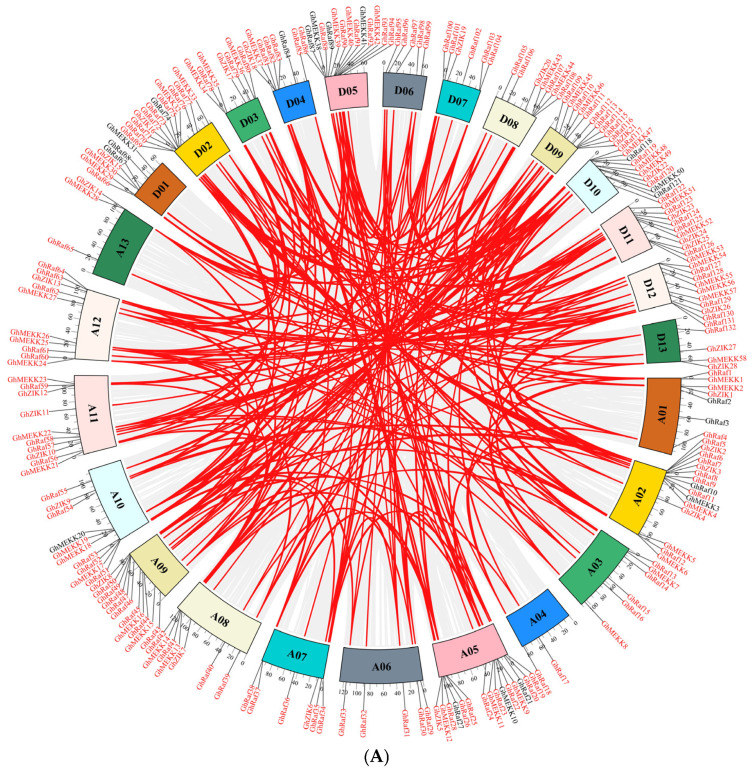

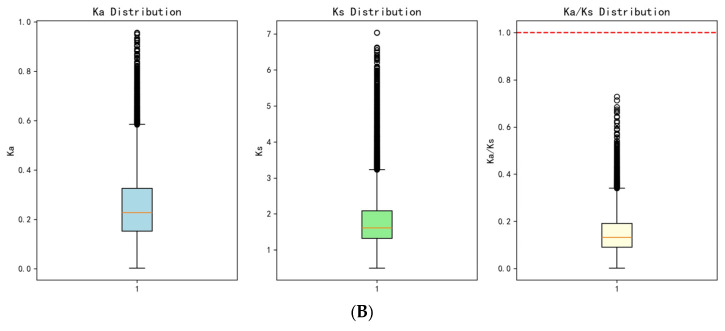
Intra-genomic synteny analysis of the MAPKKK gene family in *G. hirsutum*. The outer ring represents the 26 chromosomes of *G. hirsutum*, with chromosome numbers clearly labeled. The location of MAPKKK family members is marked on their respective chromosomal positions. The inner arcs connect gene pairs identified as syntenic. (**A**) the selective pressure analysis based on Ka/Ks ratios. (**B**) The selective pressure analysis based on Ka/Ks ratios.

To elucidate the evolutionary mechanisms underlying the expansion of the MAPKKK gene family in *G. hirsutum*, a systematic intra-genomic synteny analysis was performed. The results indicate that the expansion of MAPKKK gene family was predominantly driven by large-scale genomic duplication events. Our analysis identified a complex network of 267 syntenic gene pairs, widely distributed across all 26 chromosomes (Figure 4). Notably, pronounced syntenic blocks, harboring the majority of the syntenic gene pairs, were observed on A02, D02, A05, and D05. This distribution pattern accords with previously characterized polyploidization regions in cotton, strongly suggesting that Whole-Genome Duplication (WGD) played a decisive role in the expansion of the MAPKKK family. At the subfamily level, the Raf subfamily exhibited the most significant expansion, as reflected by the highest number of syntenic gene pairs. In contrast, the MEKK and ZIK subfamilies contained comparatively fewer duplicated pairs (Figure 4A).

The selective pressure analysis results show (Figure 4B) that there are significant differences in the distributions of Ka (non-synonymous substitution rate) and Ks (synonymous substitution rate): the median of the Ka value is about 0.2, and the fluctuation range is large, indicating that some sequences have accumulated a higher degree of non-synonymous substitution; the median of the Ks value is about 1.5–2.0, and the degree of dispersion is more obvious. Importantly, the median value of the Ka/Ks ratio is approximately 0.1–0.15, and all values are lower than 1.0, indicating that this gene family has been subject to strong purifying selection (negative selection) during evolution, and its protein-coding function is highly conserved (Figure 4B).

### 2.6. Analysis of Cis-Acting Elements in the Promoters of GhMAPKKK Genes

**Figure 5:**
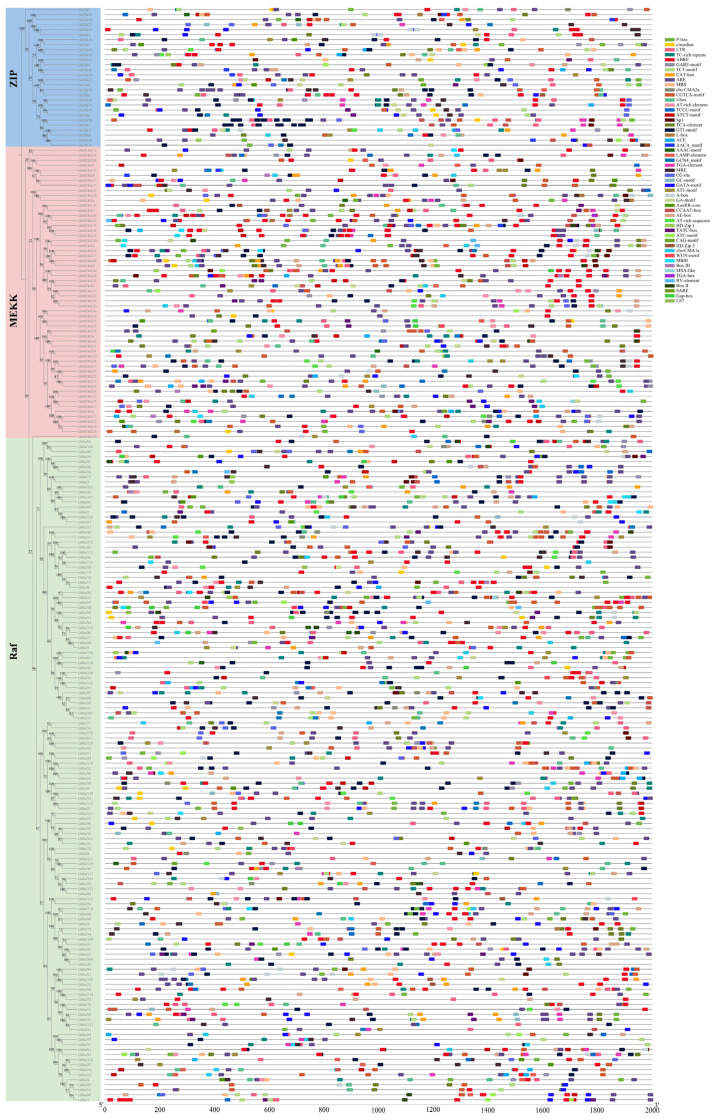
*Cis*-regulatory landscape during the evolution of GhMAPKKK genes. The left panel is the phylogenetic tree of GhMAPKKKs, showing the classification of three subfamilies (MEKK, Raf, and ZIK). The right panel shows the *cis*-acting elements located in the promoter regions of GhMAPKKKs. Different colored boxes represent distinct types of *cis*-acting elements, as indicated in icons in the upper right corner.

To explore the potential transcriptional regulation of the MAPKKK gene family, we performed a comprehensive analysis of *cis*-acting elements within the promoter regions of the GhMAPKKK genes. A total of 4063 *cis*-acting elements were identified and categorized into four major functional groups (Figure 5). The high abundance of hormone-responsive elements suggests that GhMAPKKKs play a central role in mediating defense-related plant hormone signaling. Among these, abscisic acid (ABA)-responsive element (527 ABRE) and methyl jasmonate (MeJA)-responsive element (251 CGTCA-motif) were the most abundant. This suggests that the cotton MAPKKK family is transcriptionally poised to integrate ABA, JA, and hypoxia signaling to regulate downstream defense cascades against *Verticillium wilt*. Furthermore, low-temperature responsiveness (LTR) and drought-inducibility (MBS) elements also exist. All the above indicates a synergistic regulatory mechanism operating under both biotic and abiotic stresses.

### 2.7. Gene Ontology (GO) Enrichment Analysis of GhMAPKKK Genes

**Figure 6:**
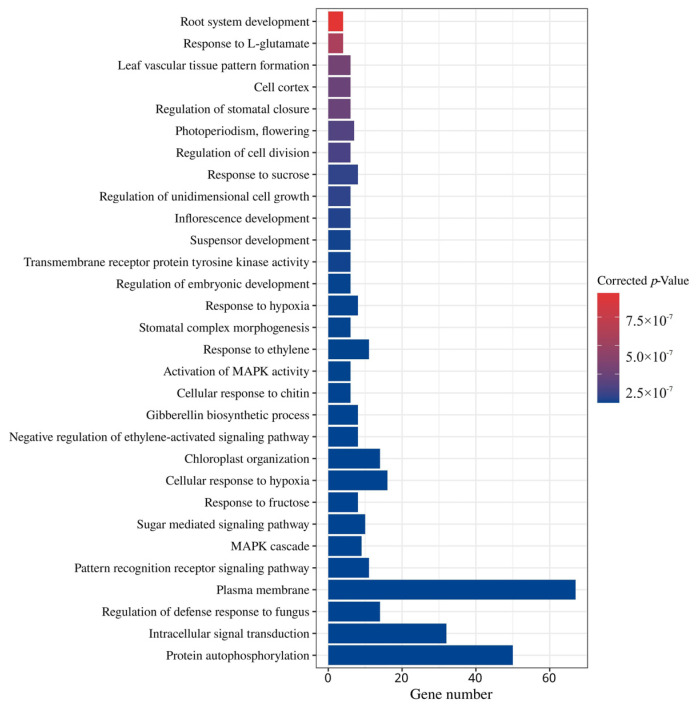
GO enrichment analysis of GhMAPKKK genes. The bar plot displays significantly enriched GO terms in the biological process category. The horizontal axis represents gene number associated with each term, while the vertical axis lists the GO terms arranged from top to bottom based on their corrected *p*-value. The color gradient from red to blue corresponds to the significance level of the enrichment, with red indicating the highest significance and blue the lowest.

The GO enrichment analysis results revealed distinct patterns among the significantly enriched terms. At the level of gene counts, the most prominent enrichment was observed in a functionally interconnected module associated with signal perception and transduction, which included “Plasma membrane”, “Regulation of defense response to fungus”, as well as “Intracellular signal transduction” and “Protein autophosphorylation”. In contrast, another set of terms—“Root system development”, “Response to L-glutamate”, “Leaf vascular tissue pattern formation”, “Cell cortex”, and “Regulation of stomatal closure”—exhibited a smaller number of enriched genes but relatively higher statistical significance (Figure 6). These terms are primarily related to plant organ development and specific signal response processes.

### 2.8. Tissue-Specific Expression Profiling of GhMEKK Genes

**Figure 7:**
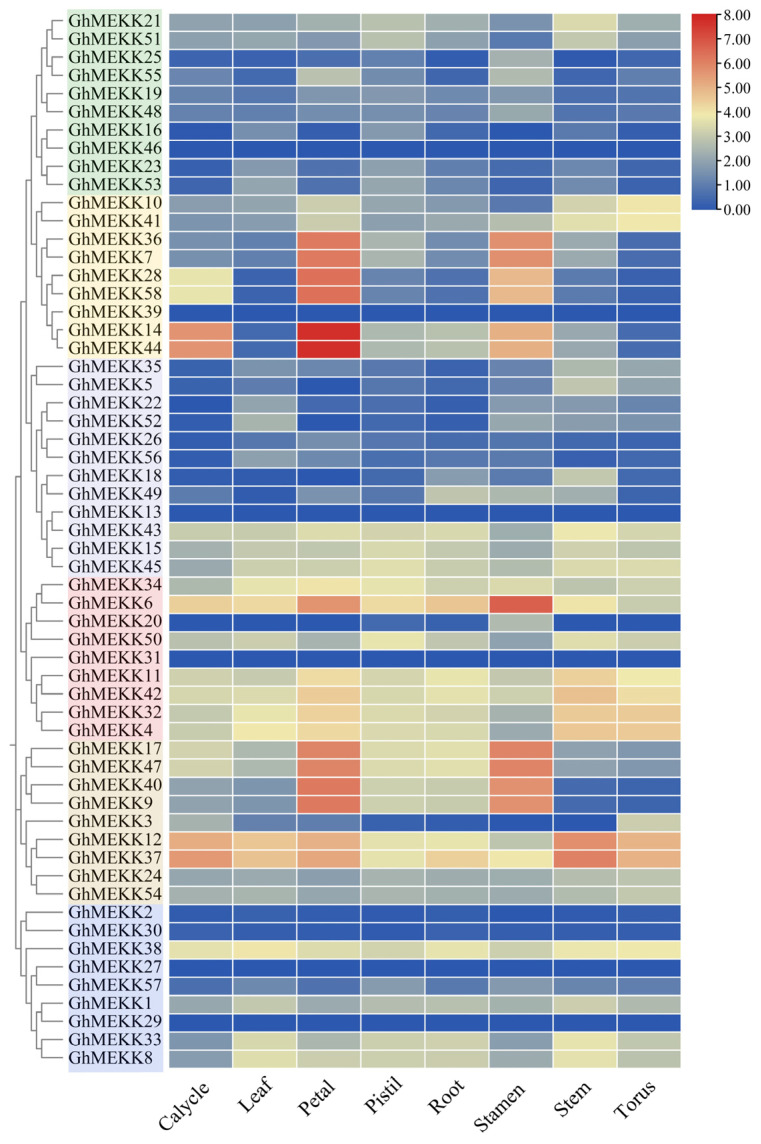
Tissue-specific expression patterns of the MEKK subfamily genes in *G. hirsutum*. The left panel is the phylogenetic tree of GhMEKKs. The right panel shows the expression heatmap of GhMEKK genes across different tissues and organs. Expression values are represented by a color gradient from red to blue, as indicated in the icon.

Expression profiling uncovered distinct tissue-specific expression patterns among GhMAPKKK genes, indicating potential functional specialization across different organs (Figure 7). In floral tissues, *GhMEKK14* and *GhMEKK44* displayed high expression in the calyx, suggesting roles in floral organ development. In anthers, *GhMEKK6* and *GhMEKK17* showed specific expression patterns, indicating potential involvement in pollen maturation and tapetal programmed cell death [28].

In vegetative tissues, *GhMEKK6* and *GhMEKK37* were root-enriched, implicating functions in root development and stress adaptation. *GhMEKK12* and *GhMEKK37* exhibited elevated expression in leaves, stems, and stipules, pointing to roles in photosynthesis, structural support, and early fruit signaling. Notably, *GhMEKK6* demonstrated significant expression across multiple tissues including pistils, anthers, and roots, while *GhMEKK37* was preferentially expressed in roots, stems, and stipules.

### 2.9. Expression Profiling of GhMEKK Genes in Response to Abiotic Stresses

**Figure 8:**
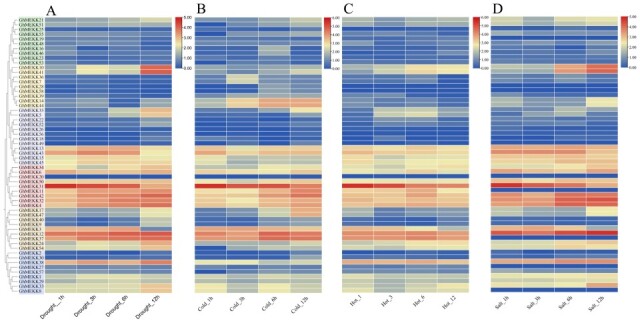
Expression profiling of GhMEKK subfamily genes under abiotic stresses. (**A**–**D**) Heatmaps display the expression patterns of GhMEKK genes under drought (**A**), cold (**B**), hot (**C**), and salt (**D**) stress treatment for 1, 3, 6 and 12 h, respectively. Expression level is represented by a color gradient from red (high expression) to blue (low expression).

To investigate the biological functions of GhMEKK genes under abiotic stress, we analyzed their expression patterns using public transcriptome data under drought, cold, hot, and salt treatments (Figure 8).

*GhMEKK14* and *GhMEKK44* showed sustained and time-dependent upregulation exclusively under cold stress, indicating a specialized role in low-temperature signaling, potentially via the CBF pathway or regulation of membrane stability genes [29,30]. In contrast, *GhMEKK11* exhibited dynamic expression across cold, heat, and drought conditions, suggesting a function as a cross-stress signal integrator [31,32]. Notably, *GhMEKK10* and *GhMEKK41* displayed synchronized induction under both drought and salt stress, while *GhMEKK37* was consistently downregulated during salt treatment. *GhMEKK31* was suppressed under all four stress conditions. These distinct expression profiles demonstrate that GhMEKK members have evolved specialized roles in responding to different environmental stresses, contributing to the precise regulation of stress adaptation in cotton.

**Figure 9:**
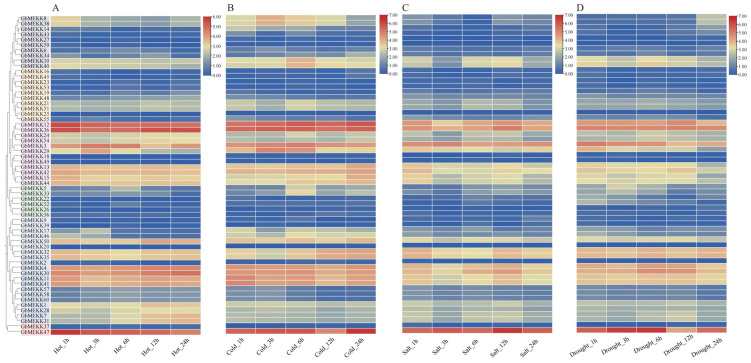
Expression profiling of GbMEKK subfamily genes under abiotic stresses. (**A**–**D**) Heatmaps display the expression patterns of GbMEKK genes under hot (**A**), cold (**B**), salt (**C**), and drought (**D**) stress treatment for 1, 3, 6, 12, 24 h, respectively. Expression level is represented by a color gradient from red to blue.

When confronted with the four abiotic stresses of hot, cold, salt and drought, the genes of the MEKK subfamily of sea island cotton generally exhibit relatively similar or have certain similarities/possess certain commonalities expression patterns. Specifically, under the regulatory effect of cold stress, *GbMEKK8/38* demonstrated more acute response characteristics and higher sensitivity. However, under the circumstances of salt stress and drought stress, its expression level is at a relatively low level. *GbMEKK12/36/47/3* showed a high expression trend under both heat stress and cold stress conditions. Notably, the expression level of *GbMEKK47* fluctuated significantly in the above four stress environments, and it is highly likely to be a dominant gene that plays a key role in multiple stress responses (Figure 9). Although the genes *GbMEKK25/18/49/20/2/37* are clustered in the same branch, from the analysis of expression level data, their expression levels are not only relatively low, but also the changing trend is not obvious.

### 2.10. Expression Profiling of the MEKK Subfamily in Response to Pathogen Stresses

**Figure 10:**
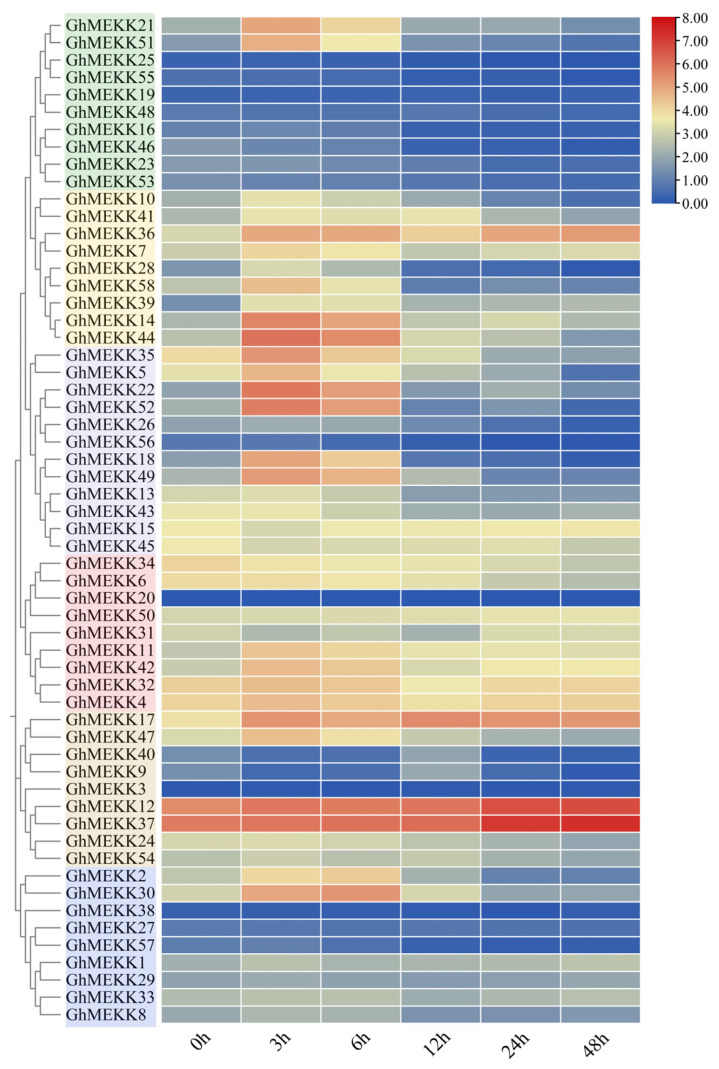
Dynamic transcriptional response of GhMEKK genes to *Verticillium dahliae* infection.

To elucidate the functional roles of MEKK subfamily genes in cotton defense against *Verticillium dahliae*, we analyzed expression dynamics in *G. hirsutum* using RNA-seq data (Figure 10). Within 48 h post-inoculation (hpi), GhMEKK genes displayed distinct temporal expression patterns. A group of genes including *GhMEKK12* and *GhMEKK37* exhibited sustained upregulation, showing progressive induction from 3 hpi, which suggests their roles as core effectors in mid-to-late immune responses. Another set represented by *GhMEKK51* and *GhMEKK21* displayed early transient responses with brief expression peaks at 3–6 hpi, potentially involved in PTI initiation. Meanwhile, genes such as *GhMEKK14*, *GhMEKK2*, and *GhMEKK30* functioned as mid-phase regulators, maintaining medium-high expression from 6 to 48 hpi, indicating their roles in signal amplification.

**Figure 11:**
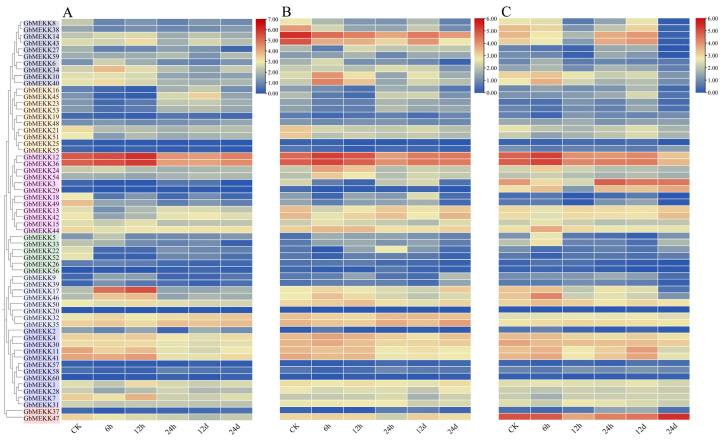
Tissue-specific defense responses of GbMEKK genes against *Fusarium oxysporum*. (**A**) Root. (**B**) Stem. (**C**) Leaf.

Analysis of *G. barbadense* under *Fusarium oxysporum* infection revealed complementary spatial expression patterns (Figure 11). A subset of genes including *GbMEKK25*, *GbMEKK55*, *GbMEKK57*, and *GbMEKK60* maintained constitutive low expression across all tissues, potentially serving as homeostasis maintainers and negative regulators of basal defense. In contrast, genes such as *GbMEKK47* and *GbMEKK54* showed tissue-specific induction with leaf-predominant expression during late infection stages. Notably, *GbMEKK17* and *GbMEKK14* emerged as core responders, demonstrating strong induction across all tissues and suggesting central roles in coordinating systemic immunity.

### 2.11. Expression Validation of GhMEKK Candidates in Response to V. dahliae Infection

**Figure 12:**
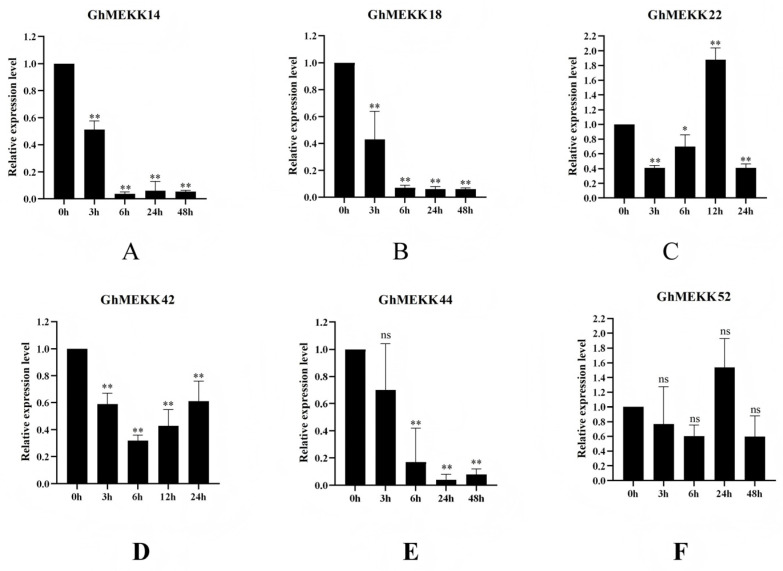
qRT-PCR validation of GhMAPKKK expression under *V. dahliae* induction. (**A**–**E**) Expression profiling of *GhMEKK14* (**A**), *GhMEKK18* (**B**), *GhMEKK22* (**C**), *GhMEKK42* (**D**), *GhMEKK44* (**E**), *GhMEKK52* (**F**) under *V. dahliae* induction for 0, 3, 6, 24, and 48 h. Gene expression was normalized to the reference gene *GhUBQ7* and calculated using the 2^−ΔΔCT^ method. Data are presented as mean ± SD from three biological replicates, each with three technical replicates. Significance levels are indicated as * (*p* < 0.05), ** (*p* < 0.01), and “ns” indicates that there is a significant difference in gene expression under the current experimental conditions, but the difference is not very pronounced, based on Student’s *t*-test.

Through integrated previous GWAS and transcriptome analysis, we identified six candidate GhMEKK genes and validated their expression patterns during *V. dahliae* infection using qRT-PCR, three distinct temporal expression profiles emerged (Figure 12) [33]. *GhMEKK14/18/44* showed sustained suppression with significant downregulation from 3 hpi, reaching minimal levels by 6 hpi. *GhMEKK22/52* exhibited biphasic regulation, initially suppressed but peaking at 24 hpi before gradual decline. *GhMEKK42* maintained stable expression throughout infection. These differential expression patterns reveal sophisticated transcriptional reprogramming of MAPK signaling components during cotton’s defense response. The sustained suppression of certain genes suggests their potential role as negative regulators, whereas the biphasic pattern indicates involvement in immune signal amplification. This precise temporal regulation demonstrates how MAPK pathway components coordinate defense activation against *Verticillium wilt* through distinct regulatory strategies.

## 3. Discussion

### 3.1. Genomic Expansion and Structural Diversification of the MAPKKK Family in Cotton

Our comprehensive genome-wide analysis identified 660 MAPKKK genes across four cotton species. The allotetraploid species (*G. hirsutum*: 218; *G. barbadense*: 230) possess more genes than their diploid progenitors (*G. arboreum*: 114; *G. raimondii*: 98), suggesting that genome duplication during polyploidization contributed to family expansion. Phylogenetic analysis classified all members into the MEKK, Raf, and ZIK subfamilies, with the Raf subfamily being the largest. Substantial variation in basic physicochemical properties (e.g., protein length, pI) was observed across members, hinting at functional diversity. Conserved motif analysis indicated subfamily-specific structural patterns. While all members retain the core kinase domain, differences were observed in regulatory regions. Notably, the predicted *cis*-acting elements in the promoter regions of MAPKKK genes—such as ABRE, LTR, MBS, and MeJA-responsive elements—show a clear correlation with their differential expression patterns under stress. For instance, cold-specifically induced genes (*GhMEKK14/44*) are enriched in LTR elements, while drought- and salt-co-responsive genes (*GhMEKK10/41*) harbor ABRE and MeJA-responsive elements, providing a plausible *cis*-regulatory basis for their expression specificity. The MEKK subfamily generally maintains motifs associated with activation loops and protein interaction interfaces. In contrast, many Raf and ZIK members show variations or absence of these regulatory motifs. These distinct structural architectures—such as C-terminal domain patterns—may reflect divergent functional specialization during evolution.

### 3.2. Functional Modularization of MEKK Subfamily Expression in Stress Responses

Our analysis reveals that GhMEKK and GbMEKK genes employ a sophisticated, modular expression strategy to coordinate responses to environmental stresses and tissue-specific requirements. Under abiotic stress, GhMEKKs exhibit clear functional specialization: *GhMEKK14* and *GhMEKK44* show cold-specific, time-dependent upregulation, potentially activating the CBF-dependent freezing tolerance pathway through cold-specific *cis*-elements; *GhMEKK11* serves as a cross-stress integrator dynamically responding to cold, hot, and drought; while *GhMEKK31* shows consistent downregulation, possibly functioning as a “signaling brake” to inhibit non-essential metabolic processes. Under salt stress, *GhMEKK10* and *GhMEKK41* display synchronized expression patterns, likely sharing ABA signaling modules, whereas *GhMEKK37* shows salt-induced suppression, possibly due to functional redundancy with the SOS pathway [34].

During pathogen infection, this functional modularization becomes more pronounced. Members differentiate into distinct functional modules based on expression kinetics and tissue distribution: (1) early warning modules (*GhMEKK51*), transiently induced during initial infection, potentially responsible for PAMP recognition and PTI signaling initiation [35]; (2) core defense execution modules (e.g., *GhMEKK12/37*, *GbMEKK17*), persistently highly expressed during mid-infection stages, coordinating systemic defense responses across tissues [36]; (3) homeostasis maintenance modules (e.g., *GbMEKK25/55*), constitutively expressed at low levels, preventing immune overactivation [37]; (4) tissue-specialized defense modules (*GbMEKK47/54*), showing leaf-predominant expression, potentially regulating organ-specific defense mechanisms. This multi-layered, modular expression pattern enables cotton to precisely and efficiently allocate defense resources with minimal energy cost, representing a key strategy in balancing growth-defense trade-offs [38].

### 3.3. MAPKKK Network in Pathogen Infection: Regulatory and Counter-Regulatory Mechanisms

Our expression profiling data revealed a temporally coordinated expression pattern of MAPKKK members during pathogen infection: some genes are transiently induced in the early stage, some genes are continuously expressed at high levels in the mid-term, and others maintain stable low-level expression. This pattern suggests that the MAPKKK family may be involved in signal amplification through orderly activation and coordinate the transition from local recognition to systemic defense [39]. In addition, some members (*GhMEKK31*) show consistent downregulation under multiple stresses, while others exhibit constitutively low expression, suggesting that negative regulatory mechanisms may be intrinsic features of this signaling network that help avoid immune overactivation, consistent with the theoretical framework of growth-defense trade-off.

The specific molecular functions of individual MAPKKK members have not been fully elucidated in this study. For example, whether the co-induction of *GhMEKK10/41* under osmotic stress depends on the ABA signaling pathway, and whether the pathogen-induced expression module is directly targeted by effector proteins [40,41,42], similar to the exploration of interacting proteins with *GhMAPKKK2* in other studies, both need to be experimentally verified.

Furthermore, the low or flat expression profiles exhibited by some GhMEKK members during pathogen infection may be influenced by various factors, such as experimental sampling conditions, post-transcriptional regulation, or the functional status of the genes (e.g., pseudogenization). These expression patterns suggest possible functional diversification and regulatory hierarchy within this family, with certain members potentially involved in signal transduction through non-transcription-dependent mechanisms. Members of our research team are currently addressing this issue, employing spatiotemporally specific sampling, protein activity assays, and gene editing approaches to further dissect and validate the underlying regulatory mechanisms of these genes.

## 4. Materials and Methods

### 4.1. Identification of MAPKKK Gene Family Members in Cotton

For the systematic identification of MAPKKK gene family members in four cotton species (*G. hirsutum* (CRI), *G. barbadense* (HAU), *G. arboreum* (CRI), and *G. raimondii* (JGI)), an integrated bioinformatics strategy was employed. All cotton genomic and protein sequence data were sourced from the CottonFGD (https://cottonfgd.org/). First, BLASTP homology searches were performed against the cotton protein datasets using known MAPKKK protein sequences from the TAIR database of *Arabidopsis thaliana* (https://www.arabidopsis.org/) as queries, with stringent thresholds set at an E-value of <1 × 10^−15^ and an identity of >50%. To complement this approach and ensure comprehensive retrieval of proteins containing the conserved kinase domain, a Hidden Markov Model (HMM) profile (PF00069) from the Pfam database (http://pfam.xfam.org/) was used to screen the same dataset using HMMER 3.3.2 software (E-value < 1 × 10^−15^). Candidate sequences obtained from both methods were merged and deduplicated. The resulting non-redundant protein set was subsequently validated using InterProScan (https://www.ebi.ac.uk/interpro/search/sequence/, accessed on 20 August 2024) and NCBI’s Batch CD-Search tool (https://www.ncbi.nlm.nih.gov/Structure/bwrpsb/bwrpsb.cgi, accessed on 20 August 2024) to confirm the presence of the characteristic protein kinase domain. Finally, key physicochemical properties—including amino acid length, molecular weight, theoretical isoelectric point (pI), and grand average of hydropathicity (GRAVY)—were predicted for all identified MAPKKK proteins using the ExPASy ProtParam online tool (http://web.expasy.org/protparam/, accessed on 20 August 2024) [42].

### 4.2. Chromosomal Localization of MAPKKK Genes

The physical chromosomal positions and gene IDs of the identified MAPKKK genes were extracted from the respective genome annotation files. Chromosome distribution maps were generated using MapChart 2.2 software and subsequently refined for presentation using Adobe Illustrator CS6 (v2020).

### 4.3. Phylogenetic Analysis

Multiple sequence alignment was performed using MUSCLE v3.8 with the following parameters: gap opening penalty = 10.0, gap extension penalty = 0.2, and the BLOSUM series weight matrix. The aligned sequences were subsequently refined using Gblocks v0.91b under default relaxed settings to remove poorly aligned positions and highly divergent regions, thereby improving alignment quality. Based on the refined alignment, a phylogenetic tree was constructed using the Neighbor-Joining (NJ) method in MEGA 11, with 1000 bootstrap replicates to assess the statistical support of branch nodes [43]. The resulting phylogenetic tree was visualized and annotated via the iTOL v6.7.6 online platform (https://itol.embl.de/). By integrating the established subfamily classification of AtMAPKKKs and their clustering patterns in the phylogenetic tree, all cotton MAPKKK members were assigned to corresponding subfamilies. Furthermore, to gain deeper insights into the evolutionary relationships of GhMAPKKKs, an independent phylogenetic analysis was conducted following the same procedure described above.

### 4.4. Conserved Motif and Gene Structure Analysis

To analyze the conserved motifs within the MAPKKK protein sequences of *G. hirsutum*, motif identification was performed using MEME Suite 5.2.0 (https://meme-suite.org/, accessed on 20 August 2024) [44]. The analysis was configured with the following parameters: maximum number of motifs = 10, motif width range = 5–50 amino acids, and site distribution model = zoops (zero or one occurrence per sequence). The resulting motif composition is summarized. Furthermore, the structural annotation information for the *GhMAPKKKs* was extracted from the whole-genome annotation file (GFF3, CRI). Finally, the conserved motif distribution and exon-intron structure of these genes were integratively analyzed and visualized using TBtools (v1.098).

### 4.5. Synteny Analysis

Intra-genomic homolinear analysis of the MAPKKK gene family was conducted in *G. hirsutum*. This analysis adopts the MCScanX algorithm implemented. The analysis parameter settings are as follows: define the minimum number of collinear genes for the same-line block as 5, set the matching score to 50, and use the e-value threshold of 1 × 10^−10^ to identify significant duplicate gene pairs. The final obtained co-line blocks and gene pair relationships were visualized using the Advanced Circos function in TBtools (v1.098). Non-synonymous mutation rate (Ka), synonymous mutation rate (Ks), and Ka/Ks value of selection pressure were obtained using the KaKs_Calculator 2.0.

### 4.6. Promoter Cis-Acting Element Analysis

The 2000 bp genomic sequences upstream of the transcription start sites of GhMAPKKK genes were extracted from the CottonFGD database (https://cottonfgd.org/, accessed on 20 August 2024) as putative promoter regions. These promoter sequences were subsequently submitted to the PlantCARE (http://bioinformatics.psb.ugent.be/webtools/plantcare/html/, accessed on 20 August 2024) database for the identification of *cis*-acting regulatory elements.

### 4.7. GO Enrichment Analysis

*A. thaliana* (TAIR10, https://www.arabidopsis.org/, accessed on 20 August 2024) homologs of the GhMAPKKKs were identified using BLASTP (https://blast.ncbi.nlm.nih.gov/Blast.cgi?PROGRAM=blastp&PAGE_TYPE=BlastSearch&LINK_LOC=blasthome, accessed on 20 August 2024) with a stringent E-value cutoff of <1 × 10^−10^. The BLASTP analysis was performed with the following parameters: max target sequences = 100, substitution matrix = BLOSUM62, gap opening penalty = 11, gap extension penalty = 1. These homologs were then mapped to GO terms. Significantly enriched GO terms in the Biological Process, Molecular Function, and Cellular Component categories were identified using a hypergeometric test, with a false discovery rate (FDR) cutoff of <0.05, |log2FC| > 1. The results were visualized using the ggplot2 package in RStudio 1.4.1717 (https://www.rstudio.com/products/rstudio/download/, accessed on 20 August 2024).

### 4.8. Expression Profiling Based on RNA-Seq Data

The RNA-seq data set used for expression analysis in this study was derived from the NCBI Sequence Read Archive database (SRA: PRJNA248163, SRP166405, https://www.ncbi.nlm.nih.gov/sra/?term=PRJNA248163, accessed on 20 August 2024), and was derived from different abiotic stress treatment experiments on upland cotton and sea island cotton seedlings [45]. Specific conditions included: treatment with 200 mM sodium chloride for salt stress; treatment with 20% (*w*/*v*) PEG-6000 to simulate osmotic drought stress; treatment of seedlings at 4 °C for cold stress; and treatment with acute heat shock at 37 °C for heat stress. All stress treatments were compared with seedlings cultured simultaneously under standard growth conditions (25 °C, normal Hoagland nutrient solution), and biological replicates were set for each treatment.

The transcriptome data analysis and gene expression quantification processing in this study followed the following process. For RNA-seq data, the raw sequencing sequences were first quality controlled by the SolexQA platform and then aligned to the reference genome using TopHat2 (v2.1.0). Transcript assembly and expression quantification were completed using the Cufflinks tool to obtain fragments per million mapped transcripts per kilobase (FPKM). Genes with FPKM > 1 were screened for subsequent analysis, and their expression levels were also normalized by log2(FPKM + 1) transformation. In all expression heatmaps shown, the color gradient represents the Z-score of the transformed log_2_(FPKM + 1) value calculated by calculating the expression of each gene across all samples to normalize expression levels and highlight relative differences. The determination threshold for differentially expressed genes (such as |log_2_FC| > 1, FDR < 0.05). Heatmaps were generated using TBtools (v1.098). The hierarchical clustering of genes and samples is based on Euclidean distance and completed using the complete linkage method.

### 4.9. RNA Extraction and Quantitative Real-Time PCR (qRT-PCR)

The upland cotton cultivar Zhongzhimian 2 (tolerant) was used as the plant material. When the seedlings reached the three-true-leaf stage, they were inoculated with *Verticillium dahliae* strain Vd592 (defoliating pathotype), the concentration of the lactarius spore suspension is 2.80 × 10^7^ spores/mL and randomly divided into two groups: pathogen-inoculated and untreated control, 0 h was treated with Sterile distilled water served as a solvent control, and other time points were treated with spore suspension. After respective treatments, root samples were collected from each group at 0, 3, 6, 24, and 48 hpi, snap-frozen in liquid nitrogen, and stored at −80 °C for subsequent RNA extraction.

Total RNA was extracted using the RNAprep Pure Kit (TIANGEN Biotech, Beijing, China). First-strand cDNA was synthesized from the purified RNA using the EasyScript^®^ One-Step gDNA Removal and cDNA Synthesis SuperMix (TransGen Biotech, Beijing, China). Gene-specific primers for quantitative real-time PCR (qRT-PCR) were designed with the Primer Premier 5 (Supplementary Table S2). The qRT-PCR assays were performed on an Applied Biosystems StepOne™ Real-Time PCR System (Thermo Fisher Scientific, Waltham, MA, USA). Each 20 µL reaction contained 10 µL of 2× TransStar^®^ Top Green qPCR SuperMix (TransGen Biotech), 0.4 µL each of forward and reverse primers (10 µM), 1 µL of template cDNA, and nuclease-free water to the final volume.

Quantitative System with UBQ7 as the internal control. The thermal cycling protocol consisted of an initial denaturation at 95 °C for 30 s, followed by 40 cycles of 95 °C for 5 s and 60 °C for 30 s. Melting curve analysis was performed from 60 °C to 95 °C with a gradual temperature increase of 0.05 °C/s after a final step of 95 °C for 15 s and 60 °C for 60 s. The relative expression levels of target genes were calculated using the 2^−ΔΔCT^ method [46]. For the differential expression analysis of RNA-seq, the criteria for differential screening (|log2FC| > 1, FDR < 0.05). Each experiment included three biological replicates, with each biological sample analyzed in three technical replicates.

## 5. Conclusions

This study provides the first systematic genomic identification of 660 MAPKKK genes across four cotton species, revealing their expansion through polyploidization-driven duplication events and their classification into MEKK, Raf, and ZIK subfamilies with distinct structural features. Multi-omics analyzes uncovered their spatiotemporal expression patterns, hierarchical regulatory networks, and functional differentiation in modular defense responses, particularly under vascular wilt pathogen stresses. These findings establish a comprehensive foundation for understanding MAPK signaling in cotton immunity and offer valuable genetic resources for future disease resistance breeding.

## Figures and Tables

**Table 1:** Identification and physicochemical properties of the MAPKKK gene family in *Gossypium* species.

Species	Gene Count	Protein Length (aa)	Molecular Weight (Da)	Isoelectric Point (pI)	Gene Family
*G. hirsutum*	28	294–735	33,509.84–83,494.35	4.66–6.66	ZIK
	58	331–1428	37,011.21–157,293.97	4.40–9.68	MEKK
	132	323–1419	36,455.53–151,585.78	4.68–9.88	Raf
*G. barbadense*	28	294–735	33,523.86–83,626.29	4.64–6.66	ZIK
	60	308–1583	34,845.39–177,408.78	4.34–9.68	MEKK
	142	270–1415	30,714.72–151,405.68	4.71–9.84	Raf
*G. arboreum*	30	336–1540	37,077.4–169,305.9	4.40–9.62	MEKK
	15	294–727	33,554.88–83,641.29	4.64–8.80	ZIK
	69	331–1401	37,366.08–151,390.64	4.69–9.84	Raf
*G. raimondii*	14	294–735	33,682.08–83,616.53	4.66–6.65	ZIK
	29	324–1390	36,030.21–153,149.16	4.37–9.84	MEKK
	55	293–1403	32,977.30–151,513.78	4.92–9.88	Raf

## Data Availability

The original contributions presented in this study are included in the article/Supplementary Materials. Further inquiries can be directed to the corresponding authors.

## Supplementary Materials

The following supporting information can be downloaded at: https://www.mdpi.com/article/10.3390/ijms27021124/s1.
